# IE-MAS: Internal–External Multi-Agent Steering for Controllable Image Captioning

**DOI:** 10.3390/e27121237

**Published:** 2025-12-07

**Authors:** Tiecheng Cai, Chao Chen, Shanshan Lin, Sibo Ju, Xiangwen Liao

**Affiliations:** 1 College of Computer and Data Science, Fuzhou University, Fuzhou 350108, China; 210310002@fzu.edu.cn (T.C.); 231016006@fzu.edu.cn (S.L.); 221010003@fzu.edu.cn (S.J.); 2 School of Computer Science and Technology, Harbin Institute of Technology (Shenzhen), Shenzhen 518055, China; cha01nbox@gmail.com

**Keywords:** controllable image captioning, multi-agent systems, information theory, affective analysis

## Abstract

Controllable Image Captioning (CIC) aims to generate coherent and semantically faithful textual descriptions of images while adhering to user-specified constraints. Existing methods have achieved promising results under individual constraints such as sentimental style or sentence length. However, they typically fail to handle and satisfy multiple constraints simultaneously, as the controls often interact and interfere with one another. To overcome these challenges, we propose Internal–External Multi-Agent Steering (IE-MAS) for CIC. IE-MAS introduces an internal multimodal steering (IMS) strategy to control affective coherence within the caption, and an external multi-agent collaboration system (EMCS) to guide visual grounding and contextual alignment. From an information-theoretic view, IMS reduces uncertainty in the generation process, while EMCS strengthens the dependency between captions and visual inputs, converting the length and sentiment constraints into information gains. Together, they produce a stable balance among semantic consistency, affective expression, and length control through an adaptive steering process that dynamically balances internal linguistic control and external perceptual grounding. Experimental results demonstrate that IE-MAS effectively coordinates multiple constraints, producing captions that satisfy the length constraint and are sentimental expressive and visually faithful.

## 1. Introduction

Image Captioning (IC) [1] aims to automatically generate coherent and semantically rich natural language descriptions for visual inputs. Controllable Image Captioning (CIC) [2] extends IC tasks by incorporating additional predefined constraints into the caption generation process, such as enforcing particular sentimental tones or regulating textual length [3,4]. The ability to satisfy fine-grained constraints over both linguistic and affective properties positions CIC as a valuable technology for diverse real-world applications, including personal multimedia recommendation systems [5] and adaptive human–computer interaction platforms [6].

The sentiment of generated captions serves as a core constraint in CIC and has attracted considerable research attention. Large language models (LLMs) achieve controllable captioning by conditioning the decoding process on explicit control signals; zero- and few-shot strategies such as prompt tuning [3] and post hoc polishing [4] provide partial control in sentiment. Recent multimodal large language models (MLLMs), including Qwen2.5-VL [7] and LLaMA3.2-vision [8], demonstrate strong cross-modal understanding and generation capabilities. However, prompt-based strategies remain inadequate for precisely identifying and modulating sentiment-relevant internal representations within these models, leading to unstable or inconsistent control effects [9]. Insufficient exploration of the latent sentiment representation space within MLLMs limits their effectiveness in achieving fine-grained controllable sentiment captioning. Thus, a key challenge lies in analyzing and adjusting the *internal* representations to enable precise and sentiment-consistent caption generation.

**Figure 1:**
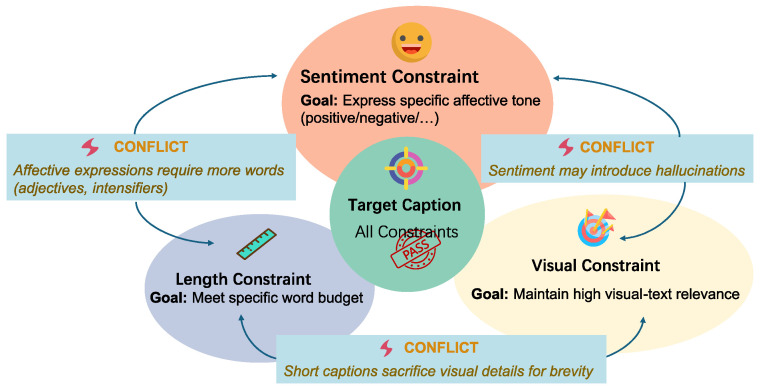
An example showing conflicts among multiple constraints in controllable image caption.

Beyond sentiment, as shown in Figure 1, CIC may incorporate *additional constraints* such as text length. Prior research on length control in non-sentiment text generation propose techniques including positional prompting and fine-tuning to guide token allocation [10,11,12], as well as post hoc sampling and rewriting methods that operate without retraining [13,14]. However, directly applying these methods in sentiment-constrained cases often distorts or even inverts the intended sentiment, revealing their fragility when multiple constraints must be satisfied simultaneously. For instance, caption length inherently affects expressive granularity: shorter captions tend to emphasize high-level visual cues, whereas longer ones enable richer affective detail but increase the likelihood of mixed or ambiguous sentiment [15]. Multi-Agent Systems (MASs) provide an alternative framework for achieving multi-constraint control in generative modeling. Recent studies have applied MASs to image captioning tasks, demonstrating that collaborative or role-specialized agents can enhance generation diversity, factual consistency, and contextual alignment [16,17,18,19]. Nevertheless, effective coordination among agents remains difficult due to conflicts arising from heterogeneous conditional objectives and potentially competing constraints [20]. Such conflicts can further lead to issues such as hallucination [17,21]. Therefore, jointly managing external constraints including sentiment, length, and visual grounding, while balancing their inherent conflicts remains an open challenge for current MAS-based captioning architectures.

Under the information-theoretic perspective, CIC is formulated as the regulation of conditional uncertainty during caption generation [22]. Given visual features and user constraints, a desired system should simultaneously minimize the conditional entropy while maximizing mutual information [23,24]. Prior studies [3,4,16] primarily operated through prompt-level control and rarely quantified or constrained uncertainty within internal representations, leading to unstable information flow and degraded caption quality when multiple constraints interact.

To address these challenges, we propose IE-MAS (Internal–External Multi-Agent Steering), a synergistic collaborative framework that integrates *internal representation steering* with an *external multi-agent coordination system*. (1) To overcome the inherent limitations of prompt-based control, we introduce Internal Multimodal Steering (IMS). IMS employs contrastive activation addition and sparse autoencoders to extract sentiment-relevant steering vectors and enhance their interpretability. By analyzing the distribution of affective correlation vectors within MLLMs and selectively injecting these vectors into the cross-modal attention layers, IMS enables fine-grained and interpretable sentiment modulation without retraining. (2) To balance competing objectives across multiple constraints, we design the External Multi-Agent Collaboration System (EMCS), comprising four cooperative agents: a Generator, a Reviewer, an Arbitrator, and a Contactor. The Contactor, serving as the mediator between external evaluation and internal steering, dynamically translates high-level feedback from the Reviewer into adaptive injection intensities for the Generator. Thus, the EMCS effectively harmonizes multiple constraints and generates coherent captions.

Experiments across distinct sentiment and length constraints show that IE-MAS consistently achieves significant improvements over strong baselines. Compared with backbone model, IE-MAS raises sentiment accuracy by over 10%, and reduces length deviation by more than 60%, while maintaining strong image–text alignment. The layer-wise analysis reveals that the deep cross-modal layers of MLLMs are most effective for fine-grained sentiment steering. Ablation studies further confirm that both the IMS and the EMCS are essential. Together, these results demonstrate that IE-MAS achieves a balanced integration of controllability, semantic consistency, and interpretability.

To summarize, our contributions are as follows.

- Internal Multimodal Steering (IMS) for fine-grained sentiment control. IMS extracts sentiment-related steering vectors and injects these vectors into the cross-modal attention layers of the base MLLM, enabling fine-grained modulation of affective tone without retraining.
- External Multi-Agent Collaboration System (EMCS) for multi-constraint controllable captioning. The EMCS balances conflicts from multiple constraints by the novel Contactor, who translates high-level external feedback into internal steering intensity. By integrating IMS and the EMCS, the proposed IE-MAS achieves adaptive coordination between internal representational control and external agent collaboration.
- Comprehensive experimental validation. Extensive experiments under different sentiment types and length settings demonstrate that IE-MAS consistently outperforms strong baselines. Layer analysis further confirms the effectiveness of internal modulation, while ablation studies verify the complementary roles of IMS and the EMCS in achieving balanced and stable Controllable Image Captioning.

The remainder of this paper is organized as follows. Section 2 reviews related work in Controllable Image Captioning, steering strategies, and multi-agent system. Section 3 illustrates the problem formulation and essential preliminaries. Section 4 introduces the proposed IE-MAS methodology. Section 5 reports experimental results and analysis. Finally, Section 6 concludes the paper and discusses future directions.

## 2. Related Work

We organize the related studies into three directions: Controllable Image Captioning (CIC), steering for CIC, and multi-agent systems. Together, these research lines trace the evolution from early constraint-based captioning to recent adaptive control frameworks for MLLMs, highlighting the progress and gaps that motivate our IE-MAS framework.

### 2.1. Controllable Image Captioning

Beyond ensuring *factual grounding* as an inherent requirement of image captioning, Controllable Image Captioning (CIC) extends conventional caption generation by introducing auxiliary constraints such as sentiment and length.

Sentiment Constraint. Early studies mainly inject sentiment signals into caption decoders through specialized embeddings or adaptive attention to balance emotional tone with visual fidelity. Mathews et al. [25] introduced SentiCap in 2016, the first RNN-based model that learns to generate captions by disentangling factual and affective words. StyleNet [26] introduces a style embedding mechanism to disentangle and control stylistic features in generated captions, while MSCap [27] incorporates multiple style representations to flexibly generate captions with diverse sentimental tones. With the rapid advancement of textual [28] and visual pretrained models [29,30], controllable captioning has been further enhanced through large-scale representation learning [3], making pretraining-based approaches the prevailing direction in affective captioning. For example, Tian et al. [31] leveraged unsupervised pretraining on large-scale unlabeled image–text data to capture both semantic and affective cues, while Wang et al. [3] employed pretraining on massive vision–language corpora to improve caption quality. Recent methods further integrate contrastive learning [32], reinforcement learning [33,34], and instruction fine-tuning [35], achieving high controllability on emotions without sacrificing linguistic quality.

Length Constraint. Beyond sentiment constraint, length constraint regulating the number of tokens/words has been explored through several paradigms. Representative approaches include length-conditioned RNNs that incorporate target length embeddings into the decoder to regulate sequence generation [36], positional encoding strategies that guide token allocation during decoding [37], and explicit length-level modeling for image captioning that achieves precise control without retraining [10].

Parallel research has explored length constraint in non-affective text generation. Representative approaches include guiding token allocation during decoding through positional prompts or fine-tuning strategies [10,11,12], as well as post hoc adjustment methods such as sampling or rewriting that modify outputs without retraining [13,14]. However, when these techniques are directly applied to CIC, they often satisfy word-length constraints at the expense of affective coherence and visual grounding.

Multiple Constraints. With the rise of LLMs in CIC, prompt engineering [38] has emerged as a pivotal technique for caption generation [39]. Recent approaches leverage prompt engineering to enable controllable captioning. Wang et al. [3] embedded learnable prompts into a pretrained captioner to enable flexible control over style and length. ConZIC [4] introduced GibbsBERT, a sampling-based non-autoregressive model that iteratively refines tokens to satisfy constraints in a zero-shot manner.

While these zero- or few-shot strategies allow partial manipulation of sentiment or length through natural-language conditioning, they remain limited to single-constraint optimization and fail to explicitly model the interplay among multiple constraints such as length, affect, and visual grounding. Moreover, although recent multimodal LLMs [7,8] have substantially expanded cross-modal reasoning capabilities, jointly satisfying multiple controllable constraints without task-specific fine-tuning remains an open challenge.

### 2.2. Steering for Controllable Image Captioning

Steering is a common method for controlling large models by adjusting internal activations or latent representations to guide model behavior [40]. The foundations of steering lie in manipulating intermediate representations to control semantics, progressively developing in recent years.

Subramani et al. demonstrated that latent steering vectors can be extracted from pretrained decoders, enabling parameter-free control of semantic attributes [41]. Building on this idea, Turner et al. formalized activation addition (ActAdd) operations that inject semantic directions into residual streams and that are effective for attribute control such as sentiment and style [40,42]. To improve robustness and interpretability, subsequent work moved toward contrastive and feature-aware methods: *Contrastive Activation Addition (CAA)* uses contrastive signals to derive more stable control directions [40], Feature-Guided Activation Additions (FGAA) introduce feature selection for more targeted interventions [43], and Sparse Steering confines steering vectors to sparse, interpretable subspaces via autoencoders [44]. Together, these developments trace a clear progression from discovering latent semantic directions to performing precise, stable, and interpretable interventions in model activations.

A few recent works have extended activation-level interventions directly to image captioning, primarily to mitigate object hallucination. Su et al. [45] proposed an activation–intervention decoding framework that integrates activation steering into the caption generation process, with empirical evaluations using CHAIR metrics demonstrating that activation-level modulation can effectively reduce hallucination without retraining. Extensions of ActAdd and CAA to image captioning confirm the feasibility of semantic correction through latent manipulation, but also expose limitations in cross-modal generalization and visual–textual alignment.

### 2.3. Multi-Agent System for Controllable Image Captioning

Multi-agent systems (MASs) have recently emerged as an effective paradigm for decomposing complex generation tasks into specialized, cooperative subtasks. Several studies explore the potential of agent-based collaboration for the image captioning generation task.

MAS for image captions. MosAIC [16] provided a multi-agent multimodal framework for cultural image captioning, where agents represent different cultural views and a central aggregator combines their outputs. MoColl [18] is a collaboration framework that mixes general and specialized agents to balance accuracy and style control. Other multi-LLM pipelines [46] adopted staged agent workflows (generation, screening, and rewriting) to improve coherence and readability. HuggingGPT [19] offered a general orchestration idea that uses an LLM controller to coordinate different expert models.

MAS for grounding captions. CapMAS [17] coordinated planner, generator, and critic agents to improve factual completeness in detailed captions and reduce hallucination. Jiang et al. [47] explored relational reasoning for image captioning and combined retrieval-augmented generation with hallucination detection to strengthen entity-level grounding. Several works focus on reducing hallucination and on grounding captions to image facts by adding retrieval, verification, or specialized critics. It shows way to make captions more faithful to the image and to user controls.

These works show that MAS architectures enhance interpretability, adaptability, and factual robustness in image captioning generation. However, their coordination mechanisms remain prompt- or response-level, focusing on surface optimization through inter-agent dialogue rather than direct internal representation control. Consequently, while MASs provide a promising foundation for multi-constraint generation, current designs neglect to directly manipulate or interpret the latent dynamics of multimodal large language models.

Table 1 summarizes representative methods in Controllable Image Captioning, activation steering, and multi-agent captioning system. In contrast to methods that typically focus on a single constraint or enforce control only at the surface text or agent level, IE-MAS combines multimodal steering with an external multi-agent coordination system to jointly handle sentiment, length, and visual grounding during caption generation. This internal–external design improves controllability under multiple constraints while preserving the generality of the underlying MLLMs.

## 3. Preliminaries

### 3.1. Problem Formulation

**Notations.** Controllable Image Captioning (CIC) aims to generate captions that are *factually grounded* in a given input image Img while satisfying predefined constraints, such as sentiment *s* [3,48] and caption length *L* [10]. The input image Img is encoded by a vision encoder ϕ [49] into a visual representation $v=\phi \left(Img\right)\in R^{d}$, the target sentiment polarity s∈S specifies the desired affective tone (e.g., positive or negative), and $L\in Z^{+}$ defines the target word budget. Prior work typically addresses only a *single* constraint at a time, neglecting potential interactions across multiple objectives. In contrast, we handle a *set* of constraints C={L,s,v}.

**Autoregressive factorization.** A generative model M produces a caption sequence $Y=\{y_{1},y_{2},\ldots ,y_{T}\}$, where $y_{t}$ denotes the token generated at time step *t* and *T* represents the sequence length. Following the standard autoregressive factorization [2], the conditional probability distribution is defined as

(1) $$\mathrm{Pr}\left(Y|C\right)=\prod_{t=1}^{T}\mathrm{Pr}\left(y_{t}|y_{<t},\;s,\;L,\;v\right),$$

where sentiment *s* dominates the token distribution at each decoding step, while length *L* and visual grounding *v* provide soft constraints to maintain caption quality. Specifically, we enforce the hard length constraint by setting T=L and prioritize sentiment alignment through internal representation steering, allowing the model to trade off between visual fidelity and affective coherence when conflicts arise.

### 3.2. Steering

Contrastive Activation Addition (CAA) and the Sparse Autoencoder (SAE) provide the foundation for steering. CAA supplies a directional control vector, while the SAE isolates a concise, interpretable basis within the activation subspace. Their integration enables precise and layer-aware sentiment modulation within MLLMs without extra fine-tuning.

Contrastive Activation Addition (CAA). CAA derives directional control vectors by aggregating activations from contrastive sample pairs, grounded in the *linear representation hypothesis* [50,51]. Let $f_{l}\left(\cdot \right)$ denote the mapping from the input to the activation space at layer *l* of a pretrained LLM. Given a positive set $D^{+}=\left\{x_{1}^{+},\ldots ,x_{n}^{+}\right\}$ and a negative set $D^{-}=\left\{x_{1}^{-},\ldots ,x_{m}^{-}\right\}$, the activations at layer *l* are denoted as $h_{l,i}^{+}=f_{l}\left(x_{i}^{+}\right)$ and $h_{l,j}^{-}=f_{l}\left(x_{j}^{-}\right)$. The CAA vector for layer *l* is computed as

(2) $$v_{l}=\frac{1}{n}\sum_{i=1}^{n}h_{l,i}^{+}-\frac{1}{m}\sum_{j=1}^{m}h_{l,j}^{-},$$

which represents the semantic direction separating positive and negative samples in the representation space.

Sparse Autoencoder (SAE). To refine the CAA vector and reduce redundancy, we adopt a Sparse Autoencoder (SAE) that transforms $v_{l}$ into a compact and interpretable representation [40,52]. Formally, the SAE first encodes the CAA vector $v_{l}$ as

(3) $$z_{l}=\mathrm{ReLU}\left(W_{E}v_{l}+b_{E}\right),$$

Then, it reconstructs a refined steering vector ${\tilde{v}}_{l}$ through the decoder:

(4) $${\tilde{v}}_{l}=W_{D}z_{l}+b_{D}.$$

The SAE is optimized by minimizing the reconstruction objective:

(5) $$L={∥v_{l}-{\tilde{v}}_{l}∥}_{2}^{2}+\lambda {∥z_{l}∥}_{1},$$

where the first term preserves semantic fidelity and the second promotes sparsity. Finally, the refined vector is injected into the model activations for controllable generation:

(6) $$h_{l}^{\prime }=h_{l}+\alpha \cdot {\tilde{v}}_{l}$$

where α controls the steering strength.

### 3.3. Multi-Agent System

Multi-agent systems (MASs) consist of multiple autonomous agents that collaborate or compete to accomplish tasks beyond the capability of a single agent [53,54]. In the context of controllable caption generation, basic MAS frameworks typically include two core types of agents: (1) Generation agents (*Generator*) produce candidate outputs according to task specifications, such as generating captions [16,17]. (2) Discrimination agents (*Evaluator* or *Reviewer*) assess the outputs with respect to predefined criteria, including factual correctness, stylistic constraints, or task-specific rules [18,46].

A typical communication among agents involves the following: the *Generator* proposes outputs, the *Evaluators* provide feedback, and optionally, decisions are passed to a mediator for selection or iteration [19]. This iterative interaction enables the system to improve output quality.

## 4. Methodology

**Figure 2:**
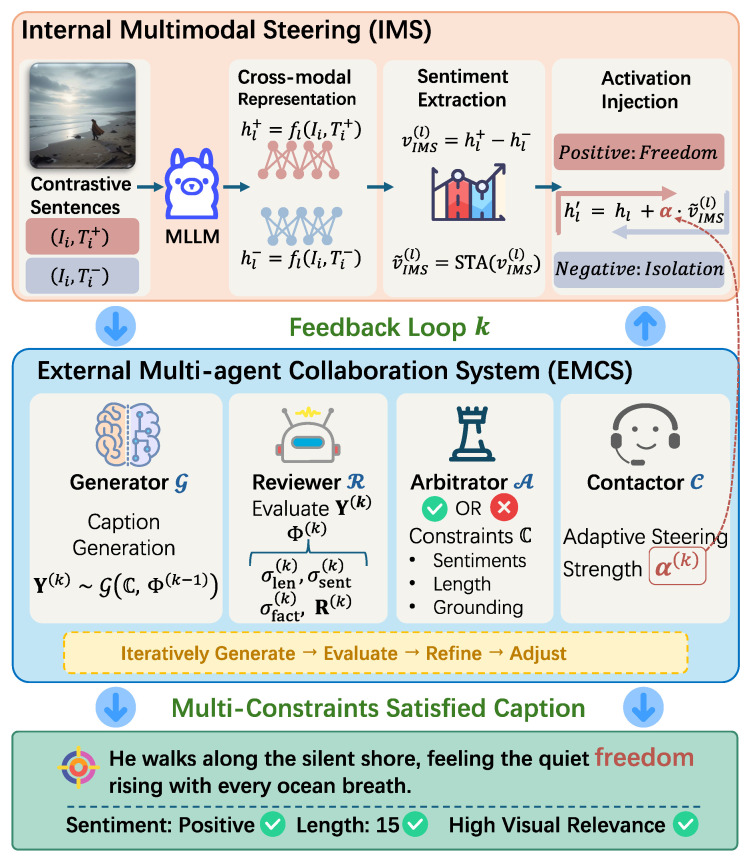
Overview of the IE-MAS framework. The IE-MAS framework integrates two complementary components: (1) Internal Multimodal Steering (IMS) extracts contrastive activation vectors from positive–negative caption pairs to steer internal representations of the multimodal large language model. (2) The External Multi-Agent Collaboration System (EMCS) consists of a Generator, Reviewer, Arbitrator, and Contactor, which iteratively generate, evaluate, and refine captions under sentiment, length, and visual constraints. The feedback loop between IMS and the EMCS enables IE-MAS to produce captions that jointly satisfy multiple control objectives.

As shown in Figure 2, IE-MAS achieves multi-constraint controllable captioning by two key components: (1) *Internal Multimodal Steering (IMS)* modifies the semantic activation patterns of a pretrained MLLM to achieve fine-grained sentiment modulation; and (2) the *External Multi-agent Collaboration System (EMCS)* performs iterative refinement to jointly satisfy sentiment, length, and image–semantic alignment. By incorporating both, IE-MAS *internally* overcomes the limitation of prompt-based control by operating at the representation level (by IMS); and *externally* reconciles multiple potentially conflicting constraints by combining steering with iterative evaluations and arbitrations (by the EMCS). IE-MAS operates purely at inference time: we do not retrain the backbone MLLM or the sentiment classifier, and all modules run as plug-in components, making it easy to integrate into existing captioning pipelines.

### 4.1. Internal Control: Multimodal Activation Steering for Cic

Existing steering studies focus on text-only LLMs, whereas steering in MLLMs differs in fundamental ways. In textual models, steering manipulates hidden activations within a *purely linguistic* representation space to adjust style or sentiment. In contrast, MLLMs jointly integrate *visual* and *textual* embeddings through cross-modal attention mechanisms, where activations simultaneously capture linguistic semantics and visual grounding. Consequently, steering in MLLMs affects not only sentiment expressed in language generation but also the model’s interpretation of visual cues.

Sentiment Direction Extraction and Refinement. To capture this joint representation, we compute the contrastive activation differences over paired image–caption inputs (I,T), rather than text-only data. The paired inputs (I,T) are extracted from a small, randomly sampled set of contrastive caption pairs. Formally, at the layer *l*, the multimodal sentiment direction is

(7) $$v_{l}^{\mathrm{IMS}}=\frac{1}{n}\sum_{i=1}^{n}\left(f_{l}\left(I_{i},T_{i}^{+}\right)-f_{l}\left(I_{i},T_{i}^{-}\right)\right),$$

where $\left(I_{i},T_{i}^{+}\right)$ and $\left(I_{i},T_{i}^{-}\right)$ represent positive and negative sentiment image–caption pairs, respectively. This cross-modal sentiment vector captures both affective and visual alignment shifts, serving as the foundation of the internal control signal within IE-MAS.

To refine $v_{l}^{\mathrm{IMS}}$ and suppress noisy activations, we draw upon the idea of Steering Target Atoms (STA), leveraging its advantage of selecting atoms in the SAE-decoupled space [9]. This enables our model to achieve finer-grained and stabler control. Specifically, we first encode $v_{l}^{\mathrm{IMS}}$ through the SAE:

(8) $$z_{l}^{\mathrm{IMS}}=\mathrm{Enc}\left(v_{l}^{\mathrm{IMS}}\right).$$

We then select the top-τ most discriminative latent dimensions (atoms) according to the absolute activation strength $|z_{l}^{\mathrm{IMS}}|$:

(9) $${\tilde{z}}_{l}^{\mathrm{IMS}}\left[i\right]=\left\{\begin{array}{cc} z_{l}^{\mathrm{IMS}}\left[i\right], & \mathrm{if}\;i\in \mathrm{TopK}\left(|z_{l}^{\mathrm{IMS}}|,\tau \right),\\ 0, & \mathrm{otherwise}. \end{array}\right.$$

Finally, the refined steering vector is reconstructed through the SAE decoder:

(10) $${\tilde{v}}_{l}^{\mathrm{IMS}}=\mathrm{Dec}\left({\tilde{z}}_{l}^{\mathrm{IMS}}\right).$$

This process isolates the most sentiment-relevant *atoms* in the latent space, effectively removing redundant and visually irrelevant components.

Activation Injection. During caption generation, the refined vector is injected into selected cross-modal representation to guide sentiment expression:

(11) $$h_{l}^{\prime }=h_{l}+\alpha^{\left(k\right)}\cdot {\tilde{v}}_{l}^{\mathrm{IMS}},$$

where α is typically a predefined hyper-parameter in existing work. In contrast, in response to other constraints, such as factual grounding, we employ an agent (Contactor C) to dynamically adapt $\alpha^{\left(k\right)}$ in the *k*-th refinement iteration.

Through this process, IMS enables the model to generate captions that convey the target sentiment with stable affective tone and preserved visual semantics, establishing a foundation for multi-constraint coordination in IE-MAS.

### 4.2. External Control: Multi-Agent Collaboration System for Cic

The external multi-agent collaboration system (EMCS) manages the controllable caption generation process. It comprises four primary agents: the *Generator* G, *Reviewer* R, *Arbitrator* A, and *Contactor* C. The system defines a communication protocol in which diagnostic feedback from the *Reviewer* and judgments from the *Arbitrator* are transmitted into actionable guidance for the *Generator*, while the *Contactor* bridges external assessments with internal steering parameters.

**Generator.** At iteration *k*, given the control specification C={L,s,v} and the guidance message from the previous step $\Phi^{\left(k-1\right)}$ (with $\Phi^{\left(0\right)}$ as the initial prompt), the *Generator* G produces candidate captions $Y^{\left(k\right)}$ via autoregressive sampling:

(12) $$Y^{\left(k\right)}∼G\left(C,\;\Phi^{\left(k-1\right)}\right).$$

**Reviewer.** For the candidate caption $Y^{\left(k\right)}$, the *Reviewer* R conducts multi evaluations, including length compliance, sentiment alignment, and image–text factual consistency. R independently assess $Y^{\left(k\right)}$ and returns diagnostic indicators, including a length deviation $\sigma_{\mathrm{len}}^{\left(k\right)}$, agreement score $\sigma_{\mathrm{sent}}^{\left(k\right)}$, factuality score $\sigma_{\mathrm{fact}}^{\left(k\right)}$, and a concise rationale $R^{\left(k\right)}$ highlighting potential errors. Formally,

(13) $$\sigma_{\mathrm{len}}^{\left(k\right)}=\left|Y^{\left(k\right)}\right|-L,$$

(14) $$\sigma_{\mathrm{sent}}^{\left(k\right)}=\mathrm{softmax}\;{\left(f_{\mathrm{sent}}\left(Y^{\left(k\right)}\right)\right)}_{s},$$

(15) $$\sigma_{\mathrm{fact}}^{\left(k\right)}=\mathrm{sigmoid}\;\left(\frac{\langle \phi_{v}\left(\mathrm{Img}\right),\;\phi_{t}\left(Y^{\left(k\right)}\right)\rangle }{∥\phi_{v}\left(\mathrm{Img}\right)∥\cdot ∥\phi_{t}\left(Y^{\left(k\right)}\right)∥}\right),$$

where $f_{\mathrm{sent}}$, $\phi_{v}$, and $\phi_{t}$ are auxiliary models. Here, $\sigma_{\mathrm{len}}^{\left(k\right)}$ quantifies the signed deviation of the generated caption’s length from the specified target *L*, $\sigma_{\mathrm{sent}}^{\left(k\right)}$ measures the alignment between the affective expression of $Y^{\left(k\right)}$ and the desired sentiment polarity *s*, and $\sigma_{\mathrm{fact}}^{\left(k\right)}$ captures the degree of semantic consistency between $Y^{\left(k\right)}$ and the visual content of the input image Img.

In addition, the *Reviewer* R generates a rationale $R^{\left(k\right)}$ identifying tokens with weak or unsupported visual grounding. These diagnostic signals are then integrated into a unified guidance message:

(16) $$\Phi^{\left(k\right)}=S\left(\sigma_{\mathrm{len}}^{\left(k\right)},\sigma_{\mathrm{sent}}^{\left(k\right)},\sigma_{\mathrm{fact}}^{\left(k\right)},R^{\left(k\right)}\right),$$

where S(·) performs structured summarization rather than naive concatenation. Specifically, R identifies the primary constraint violation and produces actionable instructions, such as “Reduce by $|\sigma_{\mathrm{len}}^{\left(k\right)}|$ words” or “Remove hallucinated tokens: [objects from $R^{\left(k\right)}$]”.

**Arbitrator.** The *Arbitrator* A enforces acceptance criteria for candidate captions based on evaluation signals. A caption is accepted before reaching the iteration limit $K_{\max}$ only if all checks pass:

(17) $$\mathrm{accept}\left(Y^{\left(k\right)}\right)\Leftrightarrow \left(\sigma_{\mathrm{len}}^{\left(k\right)}=0\right)\wedge \left(\sigma_{\mathrm{sent}}^{\left(k\right)}\geq \epsilon_{\mathrm{sent}}\right)\wedge \left(\sigma_{\mathrm{fact}}^{\left(k\right)}\geq \epsilon_{\mathrm{fact}}\right),$$

where $\epsilon_{\mathrm{sent}}$ and $\epsilon_{\mathrm{fact}}$ are sentiment and grounding thresholds, respectively. Length is enforced exactly, while sentiment and factuality are evaluated with soft margins to preserve linguistic naturalness. If no candidate satisfies all constraints after $K_{\max}$ iterations, A selects the final caption from the history $\left\{Y^{\left(1\right)},\ldots ,Y^{\left(K_{\max}\right)}\right\}$ by the priority ordering: factual grounding, sentiment alignment, and length accuracy.

During the refinement, A identifies the dominant failure mode in $\Phi^{\left(k\right)}$ and updates both the prompt and the steering strength via the *Contactor* C.

**Contactor.** The *Contactor* C serves as a communication interface between the external multi-agent system and the internal steering mechanism. It dynamically regulates the steering strength α based on sentiment deviation, thereby adapting internal control intensity based on external evaluation feedback. The existing fixed-intensity steering [9] lacks temporal adaptability: they cannot provide strong modulation in early iterations to accelerate sentiment convergence, nor can they gradually attenuate adjustments in later stages to prevent over-correction and maintain linguistic naturalness.

Formally, the adaptive steering strength $\alpha_{k}$ is updated via a non-linear mapping:

(18) $$\alpha^{\left(k\right)}=\alpha_{\min}+\left(\alpha_{\max}-\alpha_{\min}\right)\cdot {\left(1-\sigma_{\mathrm{sent}}^{\left(k\right)}\right)}^{\beta },$$

where $1-\sigma_{\mathrm{sent}}^{\left(k\right)}$ represents the sentiment deviation, indicating the divergence of the generated caption from the target affective polarity. $\alpha_{\min}$ and $\alpha_{\max}$ denote the lower and upper bounds of the steering coefficient, and β controls the smoothness of adjustment. When the deviation is large, $\alpha_{k}$ approaches $\alpha_{\max}$ for strong corrective influence; as the deviation decreases, $\alpha_{k}$ gradually converges toward $\alpha_{\min}$ for stable refinement.

Through this adaptive coordination, C integrates the external feedback signals into the internal control process, achieving balanced regulation across sentiment, length, and visual grounding. The complete iterative coordination procedure is outlined in Algorithm 1.
**Algorithm 1** The proposed IE-MAS framework
 **Require:**
Input image Img, control specification C={L,s,v}, initial prompt $\Phi^{\left(0\right)}$1:**Initialize:**  k←1, H←∅2:Calculate ${\tilde{v}}_{\mathrm{IMS}}$ by Equations (7)–(10)3:**while** $k\leq K_{\max}$ **do**4:   *#** Generator***5:   $h^{\prime }\leftarrow h+\alpha^{\left(k\right)}\cdot {\tilde{v}}_{\mathrm{IMS}}$6:   $Y^{\left(k\right)}∼G\left(C,\Phi^{\left(k-1\right)}\right)$7:   $H\leftarrow H\cup \{Y^{\left(k\right)}\}$8:   *#** Reviewer***9:   Calculate $\sigma_{\mathrm{len}}^{\left(k\right)}$, $\sigma_{\mathrm{sent}}^{\left(k\right)}$, and $\sigma_{\mathrm{fact}}^{\left(k\right)}$ by Equations (13), (14), and (15), respectively10:   Generate guidance message $\Phi^{\left(k\right)}$ by Equation (16)11:   *#** Arbitrator***12:   **if** $\left(\sigma_{\mathrm{len}}^{\left(k\right)}=0\right)\wedge \left(\sigma_{\mathrm{sent}}^{\left(k\right)}\geq \epsilon_{\mathrm{sent}}\right)\wedge \left(\sigma_{\mathrm{fact}}^{\left(k\right)}\geq \epsilon_{\mathrm{fact}}\right)$ **then**13:      **return** $Y^{\left(k\right)}$14:   **else if** $k=K_{\max}$ **then**15:     **return** $\arg\max_{Y^{\left(k\right)}\in H}(\sigma_{\mathrm{fact}}^{\left(k\right)},\sigma_{\mathrm{sent}}^{\left(k\right)},-|\sigma_{\mathrm{len}}^{\left(k\right)}\left|\right)$16:   **end if**17:   *#** Contactor***18:   **if** $\sigma_{\mathrm{sent}}^{\left(k\right)}<\epsilon_{\mathrm{sent}}$ **then**19:     Update $\alpha^{\left(k+1\right)}$ by Equation (18)20:   **else**21:     $\alpha^{\left(k+1\right)}\leftarrow \alpha^{\left(k\right)}$22:   **end if**23:   k←k+124:**end while**

### 4.3. Information-Theoretic Interpretation of IE-MAS

From an information-theoretic view, CIC can be formulated as modeling the conditional distribution p(Y|v,s,L). IE-MAS concentrates probability mass on captions that are consistent with both the image and the specific constraints, thereby reducing the conditional entropy H(Y∣v,s,L) and stabilizing generation. Formally, for fixed constraints v,s,L,

(19) $$H\left(Y|v,s,L\right)=-\sum_{Y}p\left(Y|v,s,L\right)\;\log p\left(Y|v,s,L\right),$$

where lower entropy reflects higher certainty and stronger adherence to constraints.

Conditional uncertainty and token-level entropy. During decoding, uncertainty can be estimated through token-level entropy. At refinement iteration *k*, the stepwise softmax probabilities of the generated caption $Y^{\left(k\right)}$ yield an empirical estimate of $H(Y^{\left(k\right)}|v,s,L)$. The entropy reduction between consecutive iterations is given by

(20) $$\Delta H^{\left(k\right)}=H\left(Y^{\left(k-1\right)}|v,s,L\right)-H\left(Y^{\left(k\right)}|v,s,L\right),$$

where a positive $\Delta H^{\left(k\right)}$ indicates a reduction in uncertainty and improved alignment of the conditional distribution with the target constraints from round k−1 to *k*.

IMS refines latent activations of the MLLMs to shift p(Y∣v,s,L) toward sentiment-consistent and visually grounded candidates. It follows the information bottleneck principle [55] by preserving mutual information $I({\tilde{v}}_{l}^{\mathrm{IMS}};v,s,L)$ that is most predictive of the desired caption while discarding irrelevant variability. Specifically, through the selective activation mechanism (STA in Equation (9)), IMS retains informative components in ${\tilde{z}}_{l}^{\mathrm{IMS}}$ (and thus ${\tilde{v}}_{l}^{\mathrm{IMS}}$) and suppresses noise in the latent space, thereby reducing entropy and enhancing stability in the generation process.

The EMCS further regulates the effective conditional distribution through iterative feedback. At each round, $\sigma_{\mathrm{fact}}$, $\sigma_{\mathrm{sent}}$, and $\sigma_{\mathrm{len}}$ from the Reviewer quantify mutual dependencies between the generated caption and each control signal. These signals could be viewed as proxies of measurement of mutual information between captions and corresponding constraints.

The feedback mechanism effectively reallocates probability mass toward outputs with higher joint information across modalities and constraints. We experimentally explore the correlation between mutual information and these proxies in Section 5.7.

## 5. Experiment

### 5.1. Experimental Settings

**Dataset.** Experiments are conducted on the SentiCap benchmark [25], which contains 2000 images, each annotated with three human-written captions expressing either positive or negative sentiment.

**Metrics.** To evaluate length controlability, three quantitative metrics are employed. *Mean Absolute Error (MAE)* [11] measures the average absolute deviation between the generated caption lengths and the predefined length constraint; *Exact Match (EM)* [14] denotes the proportion of captions whose lengths exactly match the prescribed constraint; and *Length Compliance (LC)* [14] reports the fraction of captions whose lengths fall within a tolerance of ±1 word. Sentiment expression is evaluated by *Sentiment Classification Accuracy (Cls.)* [27], which predicts the sentiment of the caption using a pretrained BERT-based classifier [56]. Image-semantic alignment is assessed using *CLIPScore* (*ClipS*) and *Reference CLIPScore* (*RefClipS*) [57], which are known to provide robust correlation with human judgments. Unlike n-gram-based metrics (e.g., BLEU [58], CIDEr [59]), which exhibit unreliable in zero-shot LLM outputs [60], CLIPScore directly quantifies image–caption correspondence in the joint vision–language embedding space. RefCLIPScore further incorporates human references to yield a more comprehensive evaluation of semantic consistency.

**Baseline Methods.** Our IE-MAS is compared against two backbone MLLMs: *Qwen2.5-VL (7B)* [7] and *LLaMA 3.2 Vision (11B)* [8] (without our multi-agent control). Additionally, two representative control-oriented methods are included for comparison: *ConZIC* [4], a Gibbs-sampling-based controllable captioner, and *PositionID* [11], a positional-prompting method for explicit length regulation.

**Implementation Details.** The *Generator* G is instantiated using either Qwen2.5-VL (7B) [7] or LLaMA 3.2 Vision (11B) [8]. The initial prompt $\Phi^{\left(0\right)}$ is formatted as “Please produce a single caption at *{length_constraint}* words. Use a *{sentiment_constraint}* tone.” Other default settings are shown in Table 2. All experiments are executed on a single NVIDIA A800 GPU.

### 5.2. Analysis of Main Results

Table 3 reports results concerning positive and negative sentiment constraints, respectively. IE-MAS improves both backbones and delivers the strongest overall performance with the LLaMA variant. The gains hold across all metric groups, showing that combining an external multi-agent loop with internal steering is effective regardless of the base model.

**Sentiment control.** Both IE-MAS variants raise sentiment accuracy over their bases. IE-MAS (LLaMA) keeps high accuracy for both positive and negative captions, and IE-MAS (Qwen) pushes the positive case to the top. Importantly, the negative caption collapse seen in the Qwen base is largely mitigated, showing that IE-MAS can protect affect while meeting other constraints.

**Length control.** IE-MAS (LLaMA) achieves tight length control in both sentiments, with an MAE of 0.785 and length compliance LC achieving 0.901. Other systems either drift in length or match length less reliably. The results indicate that the external feedback loop stabilizes length while the internal steering keeps edits local.

**Image–text alignment.** IE-MAS maintains strong grounding. *ClipS* and *RefClipS* are commonly better than the base models and specialized controllable captioners. The reference-based scores confirm that constraint satisfaction does not come at the cost of semantic fidelity.

**Joint optimization.** Competing methods tend to trade one objective for another (e.g., better length but weaker affect, or strong affect with poor length control). IE-MAS achieves tight length, accurate sentiment, and solid grounding at the same time, with the LLaMA instantiation offering the best balance.

### 5.3. Ablation Study: Component Contributions to Multi-Constraint Control

To evaluate the role of each component in IE-MAS, we conduct ablation experiments by removing the Internal Multimodal Steering (IMS) or the External Multi-Agent Collaboration System (EMCS) individually. As shown in Table 4, removing either component leads to clear performance degradation under both positive and negative sentiment constraints.

Without the EMCS, the model struggles to coordinate multiple constraints, resulting in a noticeably higher *MAE* and reduced *LC* scores. This indicates that external iterative feedback plays a key role in stabilizing length control and improving overall consistency. Without IMS, performance on sentiment-related metrics (*Cls.*) declines, suggesting that internal representation-level steering is essential for maintaining accurate emotional tone. Although both variants still preserve some degree of caption quality, neither can match the balanced performance of the full IE-MAS. These results collectively demonstrate that IMS and the EMCS complement each other: IMS provides fine-grained semantic control, while the EMCS ensures cooperative optimization across constraints. Their integration allows IE-MAS to achieve stable and interpretable controllable captioning.

### 5.4. Internal Layer Analysis

In contrast to text-only LLMs, MLLMs integrate both visual and textual modalities, naturally enabling affective modulation to operate across heterogeneous feature spaces but also introducing greater structural complexity. Therefore, to better understand how sentimental control works within such architectures, we deeply explore the internal behavior of MLLMs. Specifically, we take LLaMA as the backbone, which alternates between self-attention layers for linguistic encoding and cross-attention layers for vision–language fusion. Each layer thus encodes a distinct level of semantic and multimodal abstraction, providing potential sites for controllable modulation.

**Figure 3:**
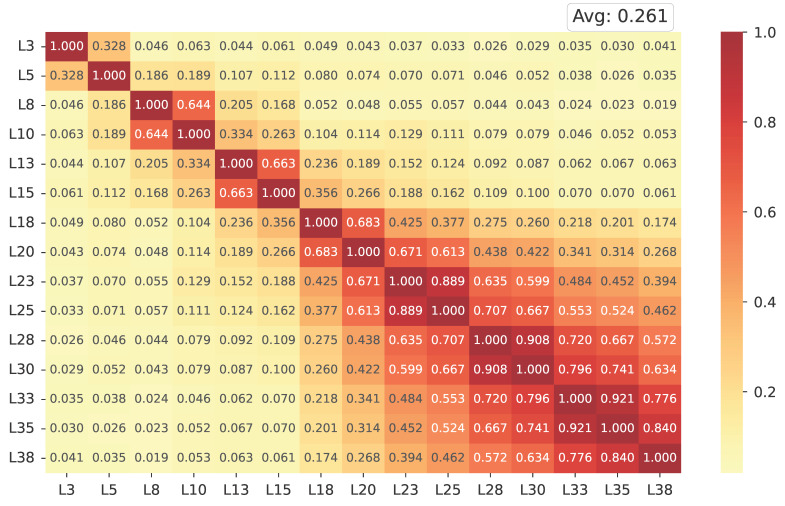
Cosine similarity heatmap of IMS vectors for distinct layers within LLaMA.

**Inter-layer similarity analysis.** Figure 3 presents the cosine similarity heatmap of IMS vectors across all Transformer layers. Each matrix entry represents the pairwise cosine similarity between the activation-based steering directions of two layers, while the average similarity (shown at the top right) summarizes their overall alignment. Both axes correspond to layer indices (L3–L38), ordered from shallow to deep, encompassing self- and cross-attention modules.

*Overall*, the heatmap reveals a low similarity (0.261), indicating that the IMS vectors extracted from different layers capture distinct, largely independent directions in the representation space. Slightly higher similarities appear among the *topmost* layers, suggesting a gradual convergence as the network approaches the output space. This layer-wise divergence demonstrates that affective information is not encoded at a single locus but refined hierarchically throughout the model depth. The observed inter-layer independence validates the rationale of specific-layer steering: by modulating selected subsets of layers rather than all of them uniformly, the model can leverage diverse yet complementary sentimental representations while preserving representational stability.

**Figure 4:**
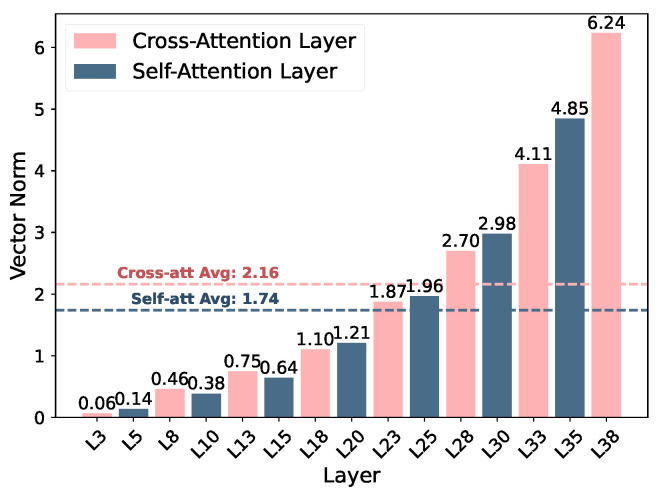
IMSvector normal distribution across layers, showing that cross-attention layers maintain higher and more variable magnitudes than self-attention layers, especially in deeper stages.

**IMS vector distribution analysis.** Figure 4 shows that vector norms steadily increase with depth, meaning that higher layers respond more strongly to steering perturbations. This reflects the progressive abstraction of semantic features, where affective modulation gains greater representational influence near the language–vision fusion and output stages.

A distinct contrast emerges between the two attention types: cross-attention layers demonstrate stronger and more fluctuating activations, peaking at 6.24 with an average of 2.16, while self-attention layers show comparatively subdued and consistent responses, with corresponding values of 4.85 and 1.74. This implies that the fusion layers, where visual and linguistic features interact, are the primary loci for effective emotional control, while self-attention layers mainly stabilize linguistic structure and syntactic coherence.

### 5.5. Sentiment-Specific SAE Feature Distribution

**Figure 5:**
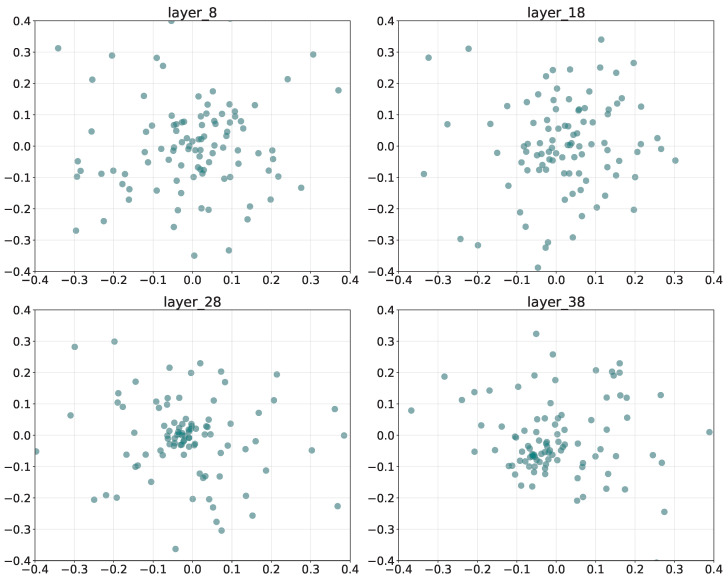
Layer-wise PCA projections of SAE-learned features, illustrating the progressive consolidation of sentimental representations from shallow to deep layers.

Figure 5 presents the PCA projections of SAE-learned features from layers L8, L18, L28, and L38 in the LLaMA backbone. The *x*- and *y*-axes correspond to the first and second principal components, capturing the major variance in the latent feature space. Each point denotes a neuron’s feature activation within the sparse representation.

The *shallow* layer (L8) exhibits a dispersed and scattered feature distribution, suggesting that affective cues remain entangled with general semantic features. By the *middle* layer (L18, L28), features begin to cluster loosely, reflecting partial disentanglement as sentiment-related neurons start to specialize. At the *deep* layer (L38), the distribution becomes compact and centralized with reduced variance, implying that sentimental information has been integrated into a unified semantic representation. Overall, the transition from dispersion to concentration reveals the hierarchical consolidation of sentimental encoding in MLLMs.

### 5.6. Sensitivity Analysis

To explore the impact of length constraints as well as the trade-off among constraints, we conduct a series of sensitivity analyses.

**Figure 6:**
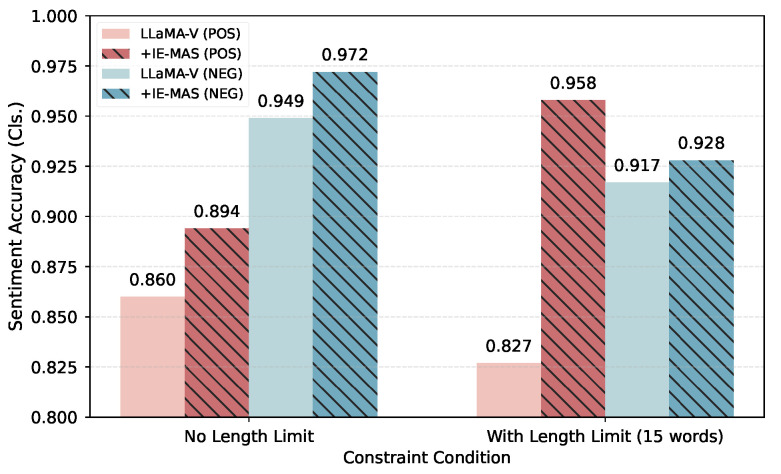
The sentiment accuracy of generated captions with or without length constraint.

**Impact of length constraint.** Figure 6 shows the impact of length constraints on sentiment accuracy under both positive (red) and negative (blue) generation settings, respectively. Each pair of bars compares the baseline LLaMA-V model (solid color) against IE-MAS (hatched pattern) under conditions with or without a 15-word length limit.

Imposing a strict length constraint consistently reduces sentiment accuracy for the baseline, indicating that restricted word budgets limit expressive flexibility and complicate the satisfaction of multiple constraints. Nevertheless, IE-MAS outperforms the baseline across all settings, confirming its controllability under various scenarios.

**Conflict between sentiment and length.** Table 5 analyzes the sensitivity of sentiment accuracy and image–text alignment across varying caption lengths.

As the length budget increases, *Cls.* declines while *ClipS* improves. This inverse relationship indicates that longer captions dilute affective focus but enhance visual grounding by incorporating more scene details. The trend highlights an inherent trade-off among length budget, emotional precision, and visual completeness.

### 5.7. Information-Theoretic Analysis

As discussed in Section 4.3, we approximate token-level entropy by averaging the stepwise softmax entropy over each caption. For each sample, the reduction in entropy equals the entropy in the first-round caption minus the one in accepted captions after iterative refinement. As a result, the average token-level entropy across the dataset decreases from 1.5245 to 1.0452 for positive captions, and from 1.7602 to 1.1254 for negative captions, yielding reductions of 0.4793 and 0.6348, respectively.

**Figure 7:**
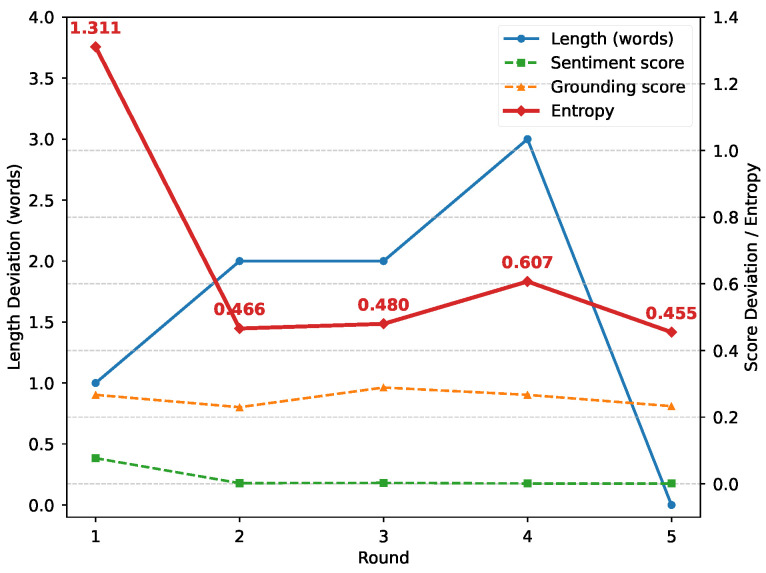
Trends of constraint deviations and approximated entropy across rounds.

Figure 7 illustrates how the empirical behavior of IE-MAS corresponds to the information-theoretic interpretation discussed in Section 4.3. In Round 1, all constraints are severely violated, resulting in high entropy and diffuse probability allocation. In Round 2, as sentiment and grounding constraints become progressively satisfied, entropy drops sharply, reflecting reduced conditional uncertainty H(Y∣v,s,L). Rounds 3–4 exhibit localized increases in entropy, where sentiment and grounding remain stable but length deviations persist, indicating transient constraint conflicts. By the final round, Round 5, all constraints are satisfied and entropy attains its minimum, corresponding to strong image–text alignment and elevated mutual information I(Y;v,s,L) under fixed conditioning inputs.

### 5.8. Qualitative Comparison

**Figure 8:**
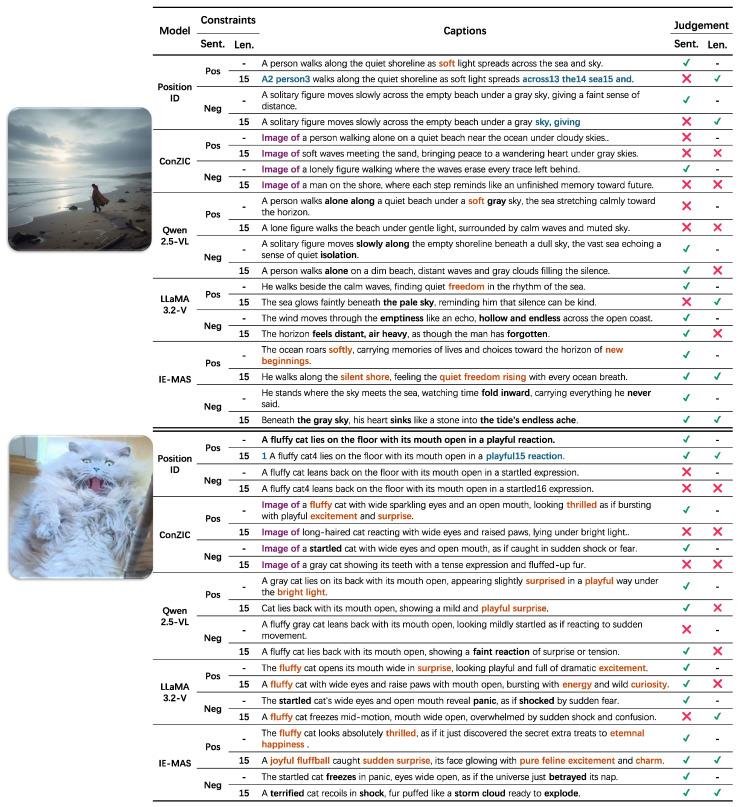
Qualitative case study illustrating caption generation under different sentiment and length constraints. IE-MAS consistently produces sentimental accurate and semantically coherent captions, even under strict length limitations. Blue indicates bugs related to “PositionID”, purple highlights the fixed prompt structure in “ConZIC”, orange marks positive sentiment words, and black marks negative sentiment words.

As shown in Figure 8, this section summarizes the results of the qualitative comparison under different sentiment and length constraints. PositionID is a position-aware embedding method that often produces captions with mixed numeric markers under sentiment conditioning. When the sequence reaches the maximum length, the generation stops directly and the caption remains incomplete. Prompt-based models such as ConZIC tend to produce fixed and repetitive sentences, often beginning with phrases like “*Image of*”. This reduces the naturalness of expression and wastes limited word space, making the generated captions less informative. *Qwen2.5-VL and LLaMA3.2-V* can express the correct sentimental tone in many cases, but it remains sensitive to length limits. When the caption length is restricted, the sentimental tone may become inaccurate or incomplete. In contrast, IE-MAS performs reliably across all conditions. It produces captions that accurately express the target sentiment, remain fluent, and preserve strong visual alignment even when the sentence length is limited. Notably, captions generated by IE-MAS exhibit richer sentimental expression, featuring vivid affective cues such as “quiet freedom rising,” “pure feline excitement,” and “the tide’s endless ache,” which convey powerful sentiment nuances beyond literal description. These results indicate that the coordinated control between internal steering and external agent modules effectively improves emotional accuracy and stability, allowing IE-MAS to balance expressive emotion with multimodal consistency.

## 6. Conclusions and Future Work

### 6.1. Conclusions

In conclusion, this paper introduces Internal–External Multi-Agent Steering (IE-MAS) for Controllable Image Captioning, a framework that coordinates internal representation-level control with external symbolic reasoning to achieve simultaneous satisfaction of multiple constraints in image captioning. From the information-theoretic perspective, IE-MAS lowers the entropy of generation under multiple constraints and increases the mutual information between captions and images, thereby conveying more image-relevant content with lower uncertainty. The internal multimodal steering strategy leverages CAA combined with SAE-based feature decomposition to manipulate sentiment-related representations. The external multi-agent collaboration system provides structured evaluation and iterative refinement through specialized agents for length, sentiment, and visual alignment, while an adaptive strength adjustment mechanism dynamically balances internal and external control based on real-time feedback.

Our comprehensive experiments demonstrate that IE-MAS effectively coordinates multiple constraints without the performance trade-offs observed in baseline methods. The ablation studies confirm that all components are essential and synergistic, with removing any single component leading to substantial performance degradation. The layer-wise analysis of CAA vectors and SAE features provides theoretical and empirical insights into sentiment representation in MLLMs.

### 6.2. Future Work

Looking forward, we plan to introduce entropy-aware adaptive decoding at inference and explore more autonomous agent interaction mechanisms, allowing uncertainty-driven dynamic communication among agents to better balance multiple constraints. We will also further investigate the internal representations of MLLMs to enhance their controllability and generalization in the field of image captioning.

## Figures and Tables

**Table 1:** Summary of representative related work on Controllable Image Captioning, steering, and multi-agent captioning systems.

Study (Ref.)	Objective	Methodology	Strengths	Limitations
2.1 Controllable Image Captioning (CIC)				
SentiCap [25], StyleNet [26]	Sentiment as a single constraint.	RNN-based captioner; style embedding module.	Pioneering sentimental CIC; explicit sentiment token control.	RNN-based small-scale models; lack multiple constraints; limited scalability and generalizability.
MSCap [27]	Multiple sentimental styles.	Multi-style captioner learning distinct style representations.	Supports affective styles with more diverse outputs.	
ConCap [3], ConZIC [4]	Multiple constraints.	Zero-/few-shot generation.	Parameter-free; flexible constraints in zero-/few-shot settings.	Visual grounding is implicit; constraint conflicts are unresolved; scalability to MLLMs is unexplored.
PositionID [11]	Length as primary constraint.	Length-aware prompts guiding token allocation.	Strong and interpretable length control	Optimizes length alone; sentiment and grounding are not modeled; fails when applying to sentimental CIC.
2.2 Steering for Controllable Image Captioning				
ActAdd [40], CAA [42]	Sentiment attribute control in text-only LLMs.	Extracts attribute directions and injects into residual streams.	Parameter-free; interpretable activation directions.	No visual grounding; steering strength fixed; ignore multi-constraint or entropy-aware control.
Activation–intervention captioning [45]	Reduce hallucination as the main constraint.	Activation interventions during decoding.	Activation-level steering can lower hallucination.	No sentiment or length control; steering intensity is not coordinated.
2.3 Multi-Agent Systems for Controllable Image Captioning				
MosAIC [16]	Culturally style captions.	Multi-agent framework.	Improves cultural relevance and diversity.	Prompt-level agent interaction; no multi-constraint trade-offs.
CapMAS [17], Planner–critic [18]	Improve factual completeness and reduce hallucination.	Planner–Generator–Critic with iterative refinement and retrieval-augmented verification.	Agent collaboration improves entity coverage and grounding.	Lacks sentimental or length control; practical complexity grows.

**Table 2:** Parameter settings for IE-MAS.

Parameter	Default Value
Min steering strength $\alpha_{\min}$	0.5
Max steering strength $\alpha_{\max}$	4
Threshold for sentiment evaluation $\epsilon_{\mathrm{sent}}$	0.5
Threshold for grounding evaluation $\epsilon_{\mathrm{fact}}$	0.25
Caption length constraint *L*	15
Number of picked atoms for activation selection τ	20
Max iterations of refinement $K_{\max}$	5
Sampling temperature for decoding	0.7

**Table 3:** Comprehensive evaluation on the SentiCap dataset concerning positive and negative sentiment constraints, respectively. The winners are highlighted in **bold**. Lower values are preferable for MAE; higher values are better for all other metrics.

Model	Sentiment	Length Control			Sentiment Expression	Image–Text Alignment	
		MAE↓	EM↑	LC↑	Cls.↑	ClipS↑	RefClipS↑
PositionID	positive	10.203	0.048	0.204	0.562	0.681	0.670
ConZIC	positive	1.632	0.129	0.452	0.477	0.799	0.745
Qwen2.5-VL	positive	3.500	0.062	0.208	0.933	0.763	0.754
**+ IE-MAS**	positive	2.871	0.091	0.282	**0.980**	0.773	0.774
LLaMA3.2-V	positive	1.942	0.176	0.441	0.827	0.802	0.802
**+ IE-MAS**	positive	**0.785**	**0.337**	**0.901**	0.958	**0.812**	**0.807**
PositionID	negative	12.274	0.042	0.143	0.498	0.648	0.678
ConZIC	negative	1.636	0.131	0.443	0.722	0.801	0.745
Qwen2.5-VL	negative	3.161	0.107	0.292	0.425	0.798	0.779
**+ IE-MAS**	negative	3.648	0.056	0.215	0.801	0.805	0.767
LLaMA3.2-V	negative	1.932	0.141	0.465	0.917	0.792	0.794
**+ IE-MAS**	negative	**0.773**	**0.363**	**0.901**	**0.928**	**0.811**	**0.804**

**Table 4:** **Ablation study**: evaluating IMS and EMCS components by removing them individually.

Model	Sentiment	Length Control			Sentiment Expression	Image–Text Alignment	
		MAE↓	EM↑	LC↑	Cls.↑	ClipS↑	RefClipS↑
IE-MAS	positive	**0.785**	**0.337**	**0.901**	**0.958**	**0.812**	**0.807**
w/o EMCS	positive	2.039	0.139	0.455	0.829	0.798	0.799
w/o IMS	positive	1.942	0.176	0.441	0.827	0.802	0.802
IE-MAS	negative	**0.773**	**0.363**	**0.901**	**0.928**	**0.811**	**0.804**
w/o EMCS	negative	1.810	0.182	0.483	0.914	0.786	0.795
w/o IMS	negative	1.932	0.141	0.465	0.917	0.792	0.794

**Table 5:** Performance under different length and sentiment constraints.

Constraints		Length Control			Sentiment Expression	Image–Text Alignment	
Length	Sentiment	MAE↓	EM↑	LC↑	Cls.↑	ClipS↑	RefClipS↑
15	Positive	0.785	0.337	0.901	0.958	0.812	0.807
20	Positive	2.358	0.099	0.443	0.887	0.821	0.798
25	Positive	5.520	0.012	0.090	0.882	0.831	0.792
15	Negative	0.773	0.363	0.901	0.928	0.811	0.804
20	Negative	2.060	0.132	0.476	0.852	0.820	0.794
25	Negative	4.952	0.028	0.098	0.791	0.831	0.792

## Data Availability

The original data presented in the study are openly available at https://users.cecs.anu.edu.au/~u4534172/senticap.html (SentiCap, accessed on 1 November 2025).
